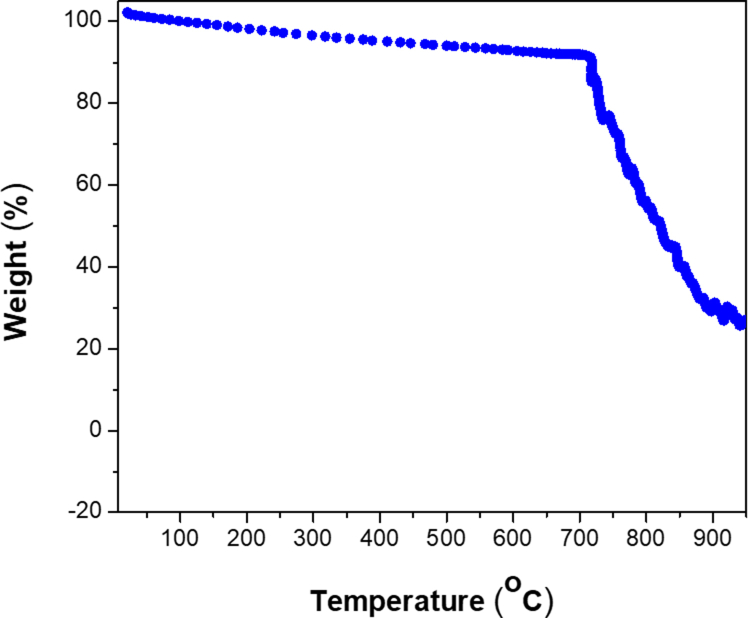# Investigation of palladium catalysts in mesoporous silica support for CO oxidation and CO_2_ adsorption

**DOI:** 10.1016/j.heliyon.2023.e18354

**Published:** 2023-07-17

**Authors:** Rola Mohammad Al Soubaihi, Khaled Mohammad Saoud, Ahmed Awadallah-F, Ahmed Mohamed Elkhatat, Shaheen A. Al-Muhtaseb, Joydeep Dutta

**Affiliations:** a Functional NanoMaterials Group, Department of Applied Physics, School of Engineering Sciences, KTH Royal Institute of Technology, Hannes Alfvéns väg 12, 11419, Stockholm, Sweden; b Virginia Commonwealth University in Qatar, Liberal Arts and Sciences Program, P.O. Box 8095, Doha, Qatar; c Department of Chemical Engineering, Qatar University, P.O. Box 2713, Doha, Qatar

**Keywords:** CO oxidation, CO_2_ adsorption, Supported catalyst, Adsorption-desorption, Palladium, Mesoporous SiO_2_ aerogel

## Abstract

The oxidation of Carbon monoxide (CO) to Carbon dioxide (CO_2_) is one of the most extensively investigated reactions in the field of heterogeneous catalysis, and it occurs via molecular rearrangements induced by catalytic metal atoms with oxygen intermediates. CO oxidation and CO_2_ capture are instrumental processes in the reduction of green-house gas emissions, both of which are used in low-temperature CO oxidation in the catalytic converters of vehicles. CO oxidation and CO_2_ adsorption at different temperatures are evaluated for palladium-supported silica aerogel (Pd/SiO_2_). The synthesized catalyst was active and stable for low-temperature CO oxidation. The catalytic activity was enhanced after the first cycle due to the reconditioning of the catalyst’s pores. It was found that the presence of oxide forms of palladium in the SiO_2_ microstructure, influences the performance of the catalysts due to oxygen vacancies that increases the frequency of active sites. CO_2_ gas adsorption onto Pd/SiO_2_ was investigated at a wide-ranging temperature from 16 to 120 °C and pressures ∼1 MPa as determined from the isotherms that were evaluated, where CO_2_ showed the highest equilibrium adsorption capacity at 16 °C. The Langmuir model was employed to study the equilibrium adsorption behavior. Finally, the effect of moisture on CO oxidation and CO_2_ adsorption was considered to account for usage in real-world applications. Overall, mesoporous Pd/SiO_2_ aerogel shows potential as a material capable of removing CO from the environment and capturing CO_2_ at low temperatures.

## 1 Introduction

The emission of toxic gases like carbon monoxide (CO) due to the incomplete combustion of fossil fuels causes significant environmental pollution. CO is a harmful gas that causes serious health problems (toxic at a concentration above 35 ppm for animals and humans due to its high affinity toward hemoglobin) [1] and impacts industrial and environmental processes [[2], [3], [4], [5]]. Furthermore, CO poisoning limits the performance of proton exchange membrane fuel cells (PEMFC) due to adsorption on the surface of platinum catalysts. Therefore, removing CO from indoor air and H_2_ streams at low temperatures (<500 °C) is important. Catalytic CO oxidation is considered one of the most effective methods to remove CO by converting it to non-toxic products such as CO_2_ and water vapor (H_2_O) [6,7]. However, the design of highly efficient and stable CO oxidation catalysts requires favorable chemical activity, stability, and selectivity in a wide temperature range (25–900 °C). On the other hand, CO oxidation generates a considerable amount of CO_2_, a greenhouse gas and a major contributor to global warming and climate change. Interest in the development of a material that catalyzes CO oxidation and acts as a sorbent for CO_2_ storage at lower temperatures has increased in recent years due to the possibility of mitigating CO and CO_2_ emissions in order to protect the environment [[8], [9], [10]].

Several catalysts have been used for CO oxidation. Namely, noble metal catalysts such as palladium (Pd) are well known for their activity toward low-temperature CO oxidation [11,12]. Catalytic Pd can exist in two stable phases (PdO or Pd^0^) depending on the oxygen concentration and reaction temperature, which enables a wide operating temperature range [13]. However, the properties of Pd catalysts are highly influenced by preparation conditions, the type and chemical nature of the support, and Pd particle dispersion within the support [[14], [15], [16]].

Silica aerogels (SiO_2_) are highly porous materials with only 10% of the bulk volume consisting of solid content, while the remaining 90% consisting of entrapped air in a highly porous network structure. In addition to their numerous applications, including catalysis [1,2], thermal insulation [[3], [4], [5]], space exploration [6], and environmental remediation [7,8], silica aerogels have also emerged in the past two decades as a viable catalyst for CO oxidation and as an adsorbent to separate contaminants from air streams [17,18]. Another important application is carbon dioxide (CO_2_) adsorption on amine-functionalized silica aerogels [19,20]. Functionalized silica aerogels possess properties for application as adsorbents in a closed-loop environmental control system [21,22]. However, a limited amount of literature reported on functionalizing silica aerogel by metal nanoparticles such as Nobel metals and their application in CO_2_ storage [[23], [24], [25], [26]]. In addition, previous studies suggest that impeding Pd nanoparticles in porous matrices such as aerogels, carbon frameworks, and metal-organic frameworks (MOFs) directly influences the catalytic activity [23] as well as gas storage, separation, and utilization [23,27,28]. Therefore, a silica support is used due to its well-known thermal stability and resistance to sintering at high temperatures [29,30]. For example, Pd/SiO_2_ catalyst is a well-known stable catalyst for low-temperature CO oxidation in the range of 150–250 °C [31]. Achieving high palladium dispersion within a silica-based support is challenging due to many factors, including high hydrophobicity and poor ion exchange capacity. Research in this area could aid the development of future heterogeneous Pd catalysts for a wide range of reactions besides CO oxidation [32]. We recently reported mesoporous silica aerogel-supported palladium catalyst (Pd/SiO_2_) for low-temperature CO oxidation under ignition/extinction cycles with excellent stability [12]. The idea of using the same nanomaterials that serve as a catalyst for CO oxidation as a sorbent was recently reported by several groups [[8], [9], [10],^33^]. However, some of the materials used in these studies suffer poor stability at higher temperatures.

This work serves as an investigation of a potential improvement to the pathways used in the removal of toxic gases (namely CO) from the environment by converting them to CO_2_. The main strategy is based on silica aerogel-supported Palladium catalytic materials for CO oxidation and CO_2_ adsorption at lower temperatures. Mesoporous Pd/SiO_2_ aerogels were prepared and characterized. Then, they were utilized for the CO conversion to CO_2_ and CO_2_ adsorption at ranges of temperatures and pressures. For this purpose, the adsorption-desorption experiments were conducted at different temperatures and pressures and correlated with the standard adsorption models. Furthermore, effect of the presence of humidity in the gas mixture on the CO oxidation and CO_2_ adsorption processes was considered. This study presents a strategy for studying catalytic materials for CO oxidation and understanding their CO_2_ adsorption capabilities.

## 2 Experimental

### 2.1 Materials

The materials used in this study included Tetraethoxysilane (TEOS, Sigma- Aldrich, 98%), Aluminum Chloride Hexahydrate (AlCl_3_·6H_2_O), Diethylmethylamine (C_5_H_13_N, Sigma Aldrich), Palladium Chloride (PdCl_2_, Sigma-Aldrich). Ethanol (ethanol-water azeotrope mixture, Sigma-Aldrich, 4.4% water, and 95.6% pure ethanol) was used as a solvent in the gelation process and as a supercritical fluid in the drying process. All chemicals are used without further purification.

### 2.2 Synthesis and preparation

**Fig. 1:**
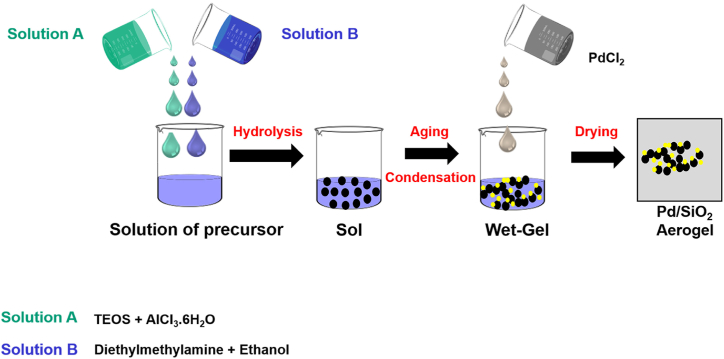
Schematic representation of the palladium/silica (Pd/SiO_2_) aerogel synthesis procedure.

Palladium supported on mesoporous SiO_2_ Aerogel (Pd/SiO_2_) catalyst with 5 wt% Pd loading was synthesized via the Sol-Gel method [12]. Silica and supported metal silica aerogel samples were prepared using the sol-gel synthesis method reported elsewhere [34]. The synthesis is in this study followed an acid-catalyzed synthesis route. A silica precursor (TEOS) is dissolved in ethanol with water (in a stoichiometric amount) in a typical synthesis. A small amount of AlCl_3_·6H_2_O acid acts as a catalyst for hydrolysis and condensation reactions [34], and is mixed with diethylmethylamine solution to adjust the pH to 8.5. The TEOS/ethanol/H_2_O mole ratio was 1/9.1/1.4 for the gels with the highest TEOS concentration, while the concentrations of AlCl_3_ and diethylmethylamine in the gelation solution were 1.05 mmol/1 and 1.376 mmol/1, respectively. The liquid mixture is converted into alcogel in less than half an hour. The gel is aged, then dipped in the metal precursor (3.0 mmol/l concentration equivalent to 5% Pd) for two days to ensure solvent exchange with the metal precursor, and finally dried in ethanol under supercritical conditions [35]. The dried metal-supported silica aerogel was removed from the autoclave and powdered for further catalytic experiments, adsorption studies, or characterization. The dried samples are highly porous materials for catalytic activity or adsorption studies. The complete synthesis steps are summarized schematically in Fig. 1.

### 2.3 Characterization studies

Samples were characterized by X-ray diffraction (XRD) using (X'Pert PRO X-ray diffractometer, USA) powder diffractometer with Cu-K_α_ radiation (λ = 1.54060 Å) operated at 40 kV and 30 mA, the 2θ range from 10° to 80° with a step size of 0.01° and acquisition time of 0.5 s. X-ray photoelectron spectroscopy (XPS; Omicron Nanotechnology, Germany) with a monochromatic Al K_α_ radiation (energy = 1486.6 eV) working at 15 kV was used to study the surface states of the Pd/SiO_2_ aerogel. The base pressure (1.33 × 10^−7^ Pa) rose to 1.33 × 10^−6^ Pa during analysis. Casa XPS software was used in data analysis and peak fitting. XPS spectra were calibrated to the C1s feature at 284.6 eV. Temperature programmed reduction (TPR) profile was studied using Micromeritics Autochem II with a flow rate of 50.04 cm^3^ STP/min. Thermogravimetric analysis (TGA) was performed using a Thermogravimetric analyzer (TG 2100D, Analytical Technologies Limited, Shanghai, China) analyzer from (23 °C–800 °C) at a ramp rate of 10 °C/min and a flow rate of 100 cm^3^/min in a nitrogen atmosphere. N_2_ adsorption-desorption isotherm at 77 K was employed to investigate the surface area of Pd/SiO_2_ aerogel. High-resolution transmission electron microscopy (HR-TEM) measurements were carried out using a JEM2100F field emission transmission electron microscope (JEOL USA, Inc.) operating at an accelerating voltage of 200 kV. Fourier transform infrared (FTIR) spectroscopy was performed using the FTIR650 spectrometer with LA-025-1100 universal ATR unit (Labfreez Instruments (Hunan) Co., Ltd., Hunan, China).

### 2.4 Catalytic activity tests

Catalytic activity tests were carried out in a quartz fixed-bed tube flow reactor (i.d. = 10 mm), loaded with 20 mg of catalyst sandwiched between pieces of quartz wool. Before experiments, catalysts were calcinated at 450 °C for 1 h under air. CO oxidation catalytic experiments were conducted at atmospheric pressure (1 atm) in the temperature range of 25–600 °C with a heating rate of 10 °C/min. The flow rate of the feed gas mixture was 100 cm^3^/min, corresponding to a weight hourly space velocity (WHSV) of 38,000 cm^3^/g h. The feed gas mixture consisted of a 3.6% CO and 20% O_2_ mixture balanced with He gas (cylinder size 50 L, pressure 1500 MPa, valve type BS3, Buzwair gases, Doha, Qatar). The catalytic performance of investigated catalysts was assessed in the conversion of CO to CO_2_ and measured as a function of reaction temperature based on the CO concentration in the feed gas and the product gas. The CO conversion efficiency was calculated following Equation (1) as below [36]:

(1) $$CO\;conversion\;\left(\%\right)=\frac{{\left[CO\right]}_{in}-{\left[CO\right]}_{out}}{{\left[CO\right]}_{in}}\times 100\%$$

where [CO]_in_ and [CO]_out_ are the CO concentrations in the reaction feed mixture and the produced gas, respectively.

### 2.5 Adsorption studies

The CO_2_ storage studies were performed using a Magnetic suspension balance (MSB), (Rubotherm Präzisionsmesstechnik GmbH, Germany) that measured the adsorption/desorption isotherms of CO_2_ gas at different temperatures. This instrument had a microgram sensitivity in its sample weight measurements. The procedure of measuring the adsorption and desorption isotherms is explained in detail elsewhere [37].

## 3 Results and discussion

### 3.1 Characterization study

**Fig. 2:**
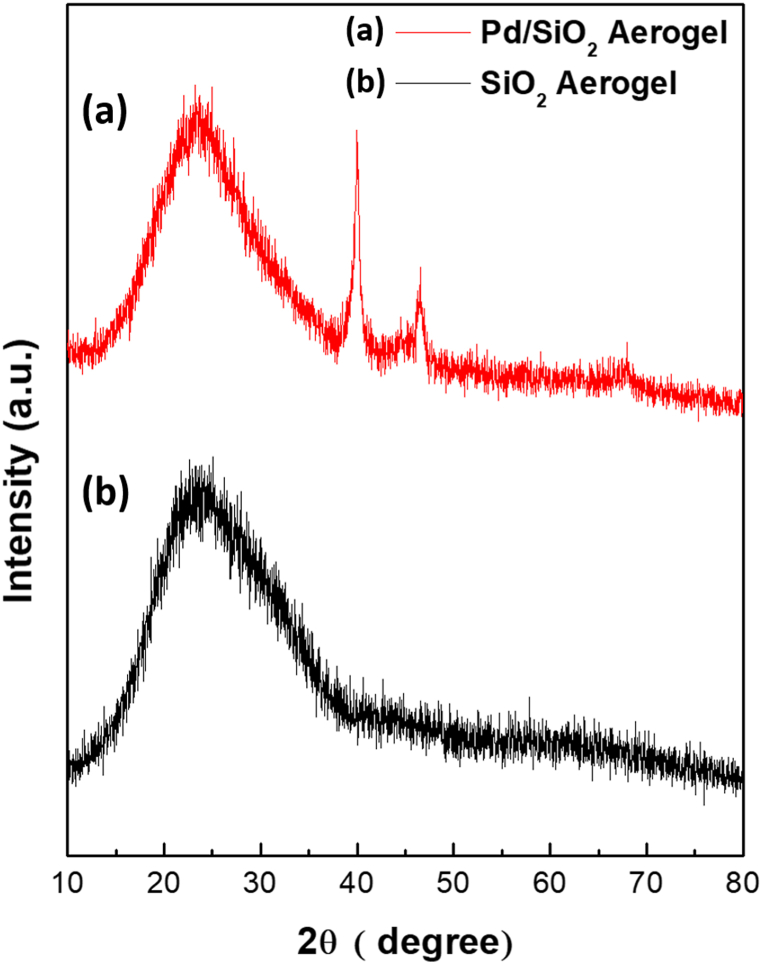
X-Ray Diffraction (XRD) patterns of (a) 5% palladium/silica (Pd/SiO_2_) aerogel and (b) native silica (SiO_2_).

Fig. 2 shows the XRD pattern of a fresh Pd/SiO_2_ aerogel sample (Fig. 2(a)) compared to that of native SiO_2_ aerogel (Fig. 2(b)). The patterns confirm the presence of amorphous mesoporous nano-silica in both samples, which is consistent with (JCPDS card no. 16-0380) [38]. However, the XRD patterns of Pd/SiO_2_ aerogel showed sharp diffraction peaks located at the 2θ angles of 40.1°, 46.7°, and 68.2° beside the usual SiO_2_ peak. These peaks were identified to correspond to the (111), (200), and (222) planes, which are consistent with crystalline face-centered cubic (FCC) palladium (JCPDS card, File No. 46-1043) [39], suggesting the formation of metallic Pd nanoparticles.

**Fig. 3:**
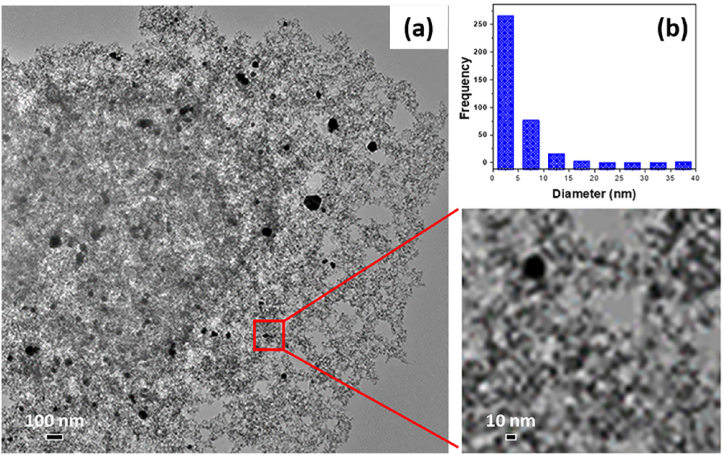
(a) Transmission Electron Microscope (TEM) of 5% palladium/silica (Pd/SiO_2_) aerogel catalyst with Pd (black) within the matrix, inside pores, and on the surface of the mesoporous silica, (b) Pd nanoparticles size distribution histogram.

The TEM micrographs of Pd/SiO_2_ aerogel (Fig. 3(a)) reveal the presence of a mesoporous SiO_2_ aerogel framework with a large number of small Pd particles (2-3 nm) dispersed homogeneously within the SiO_2_ pores and framework, as well as several large Pd surface particles (20–80 nm) on the surface of the silica framework (Fig. 3(a and b)), which is typical for mesoporous silica structures [40,41].

**Fig. 4:**
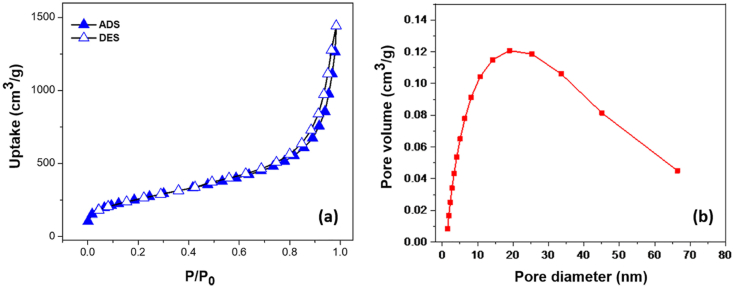
(a) N_2_ adsorption-desorption characteristics of 5% palladium/silica (Pd/SiO_2_) aerogel catalysts isotherms at 77 K, (b) pore diameter distribution in 5% palladium/silica (Pd/SiO_2_) aerogel.

The adsorption-desorption isotherms of N_2_ on Pd/SiO_2_ aerogel at −196 °C (77 K) (Fig. 4(a)) reveal that the Pd/SiO_2_ aerogel has a large Brunauer−Emmett−Teller (BET) specific surface area of 1114 m^2^/g, which is larger than the specific surface area of native aerogel (∼478 m^2^/g) [12] and is consistent with previously reported literature and typical of hierarchical mesoporous silica structure with well-dispersed Pd nanoparticles on the surface and in mesopores [42]. The isotherm shows a marked uptake of type IV according to the International Union of Pure and Applied Chemistry (IUPAC) classification due to the capillary condensation phenomenon, where a small H2 hysteresis loop at p/p_0_ > 0.75 indicates mesoporous microporous characteristics [37]. The isotherm is also characterized by the increment of adsorption at p/p_0_ > 0.9, which is caused by larger mesopores typical for mesoporous materials [38]. The Pd/SiO_2_ aerogels show higher uptake due to the high specific area and a hysteresis loop due to the large pore volume. The pore diameter distribution of Pd/SiO_2_ aerogel (Fig. 4(b)) reveals a continuous distribution with a pore diameter of 2–60 nm and different pore sizes**,** which is atypical for aerogels [40]. The presence of excess palladium during synthesis would increase the number of micropores, resulting in a large surface palladium particle outside the pores.

**Fig. 5:**
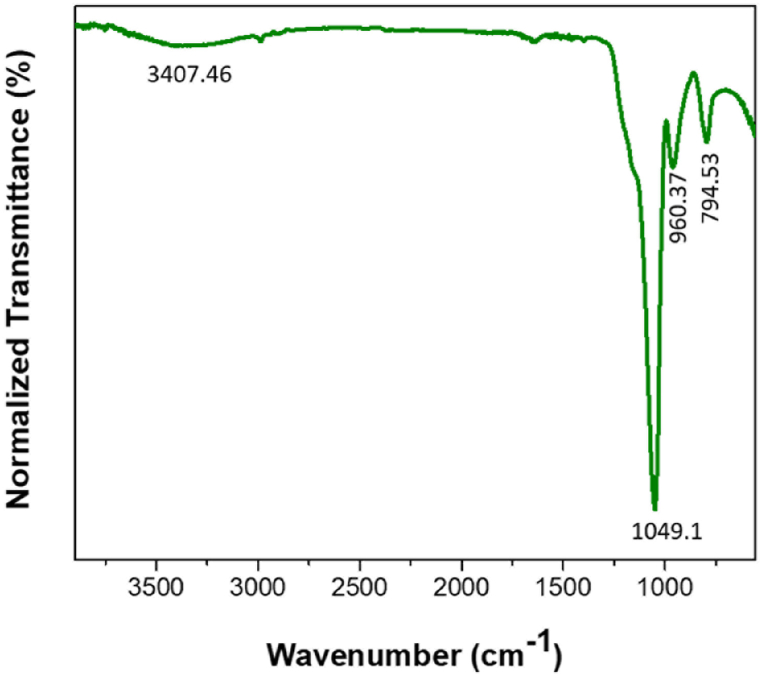
Fourier transform infrared (FTIR) spectra of 5% palladium/silica (Pd/SiO_2_) aerogel.

FTIR spectrum of Pd/SiO_2_ aerogel reveals signature of stretching vibrations of Si–O–Si bridges. The broad absorption peak centered at ∼3408 cm^−1^ was assigned to Si–O–H stretching vibrations, while the peak centered at ∼960 cm^−1^ was assigned to Si–OH stretching vibrations (Fig. 5). By comparing the intensity of the peak in the ∼2800–3500 cm^−1^ region with that of native SiO_2_ aerogel [12], the results revealed that the presence of palladium in the Pd/SiO_2_ aerogel sample caused a 10 cm^−1^ shift toward a lower wavenumber, suggesting an interaction of the Pd with the underlying SiO_2_ framework. The presence of silicon hydride groups in the silica framework induces *in-situ* reduction of palladium species to metallic particles, and the confined space in pores could be responsible for the formation of small palladium nanoparticles [43].

**Fig. 6:**
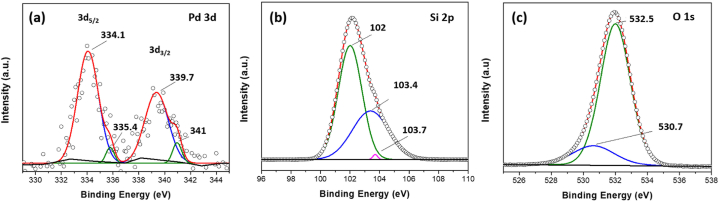
X-ray photoelectron spectroscopy (XPS) spectra of (a) Pd 3d with Pd^0^ (blue) and Pd^2+^ (green) deconvolution peaks, (b) Si 2p with *-*Si-O-Si- (green), stochiometric SiO_2_ (blue), and -Si-OH (magenta) deconvolution peaks, and (c) O 1s with Si–O–Si- (green) and Pd–O (blue) deconvolution peaks in 5% palladium/silica (Pd/SiO_2_) aerogel.

XPS of Pd 3d spectra (Fig. 6(a)) for the synthesized palladium/silica (Pd/SiO_2_) aerogel confirms the existence of metallic palladium (Pd^0^) with the spin-orbital splitting of 5.2 eV and palladium oxide (Pd^2+^). The XPS spectra showed a small shift of the peaks to lower binding energy due to electron transfer from the Pd to SiO_2,_ suggesting a strong interaction between the silica support and palladium [44]. XPS spectra of Si 2p (Fig. 6(b)) can be deconvoluted into two major peeks assigned to the -Si-O-Si- functional group, stochiometric SiO_2_, and a small -Si-OH peek. The spectra showed that the SiO_2_ signal is shifted 1.6 eV towards higher binding energies from the main Si 2p, which could be attributed to differential charging between the SiO_2_ host and the palladium particles [45,46]. The deconvolution of oxygen O 1s spectra (Fig. 6(c)) revealed two Pd/SiO_2_ aerogel peaks corresponding to oxygen vacancies or SiO_x_ and stoichiometric SiO_2_. This agrees with the previous literature on O 1s binding energies, which confirm the presence of palladium oxide or (Pd^2+^) species [47]. One of our previous works introduced a detailed XPS analysis of the Pd^2+^ species before, during, and after CO oxidation [36].

**Fig. 7:**
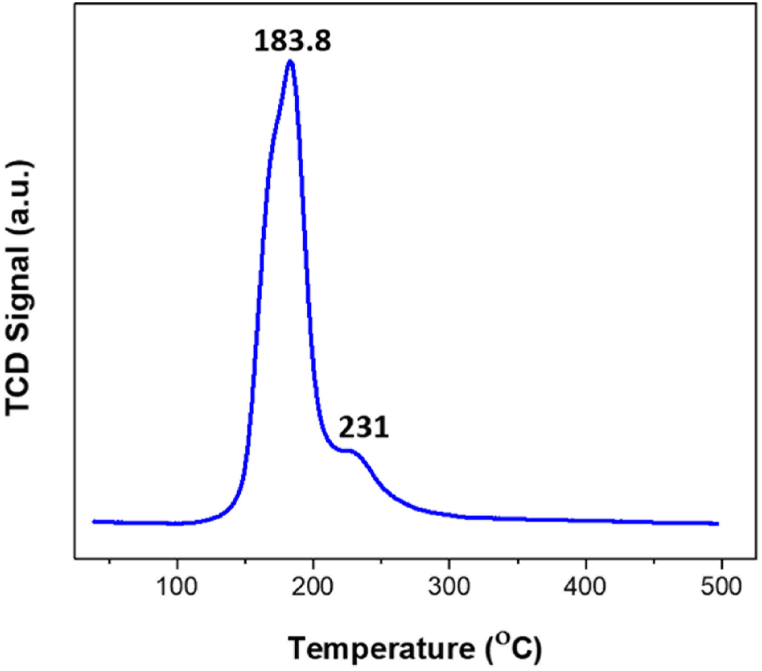
Temperature programmed reduction (TPR) profile of 5% palladium/silica (Pd/SiO_2_) aerogel.

The positive TPR peaks of Pd/SiO_2_ aerogel (Fig. 7) indicate that bulk PdO_x_ species were reduced to Pd^0^ [48,49]. The large reduction peak is attributed to the reduction of highly dispersed PdO_x_ species caused by the synthesis step and atmospheric exposure of the sample to air before reducing metallic Pd [39,50]. The small peak is attributed to Pd^2+^ in the SiO_2_ phase [51]. Furthermore, the absence of negative peaks in the TPR profile at low temperatures indicates the absence of partially reduced palladium at room temperature. These results agree with previous studies [52] and the XPS results.

**Fig. 8:**
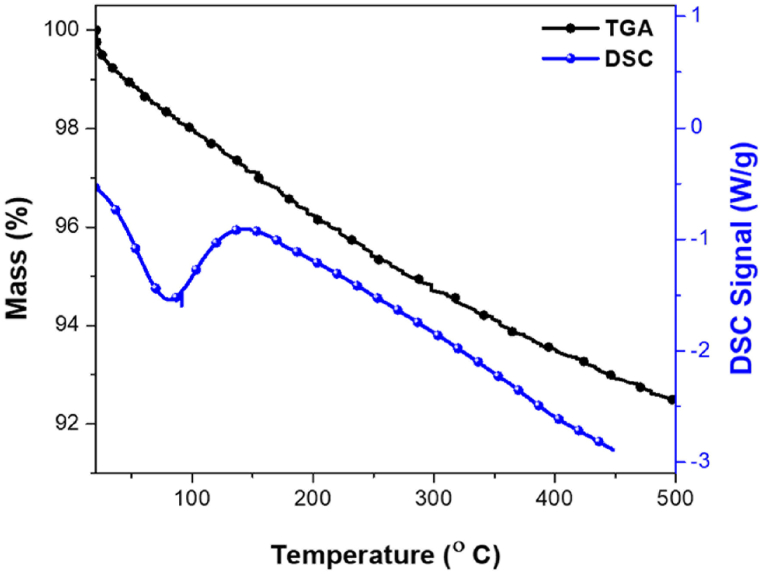
Thermal gravimetric analysis-differential scanning calorimetry (TGA-DSC) curves of 5% palladium/silica (Pd/SiO_2_) aerogel heated in the N_2_ atmosphere with a 10 °C/min heating rate.

Pd/SiO_2_ catalysts show relative thermal stability until their thermal degradation at ∼700 °C (Fig. S1). Pd/SiO_2_ aerogel has an initial mass loss (about 2%) in the low-temperature stage (25–125 °C) due to the removal of adsorbed moisture, the evaporation of the solvents, and the removal of the remaining unreacted alkoxy groups; this is verified by the DSC endothermic peak in the stage from room temperature up to 125 °C (Fig. 8). However, upon increasing the temperatures, the samples showed another mass loss (5%) accompanied by a DSC exothermic peak in the stage of 125–250 °C, which could be attributed to the oxidation of palladium along with a rapid increase in condensation of silanol groups (reduction of the silica surface) and the formation of the interface between the palladium and silica support [5].

### 3.2 Catalytic conversion of CO

**Fig. 9:**
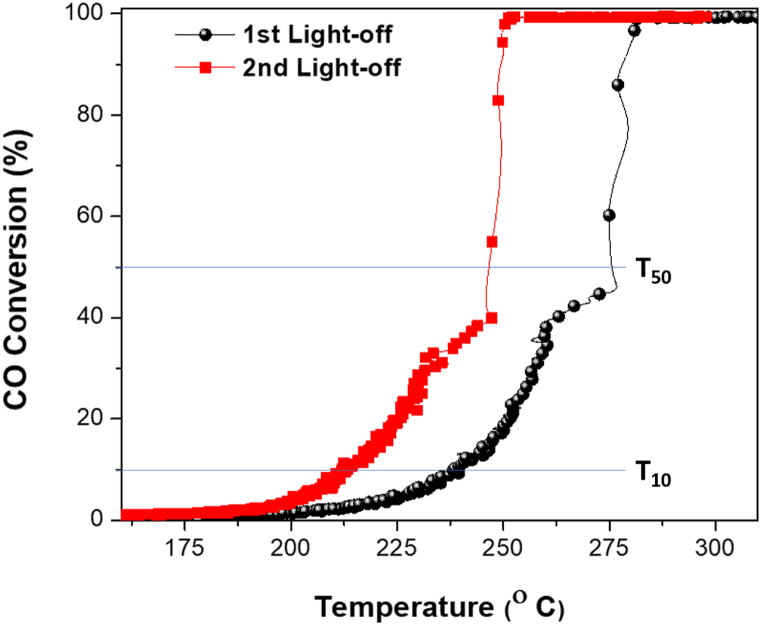
Carbon monoxide (CO) conversion over 5% palladium/silica (Pd/SiO_2_) aerogel as a function of Reaction temperature (20 mg catalyst weight, 10 °C/min heating rate, and 100 cm^3^/min flow rate).

CO conversion with Pd/SiO_2_ aerogel catalysts as a function of the reaction temperature is presented in Fig. 9. CO conversion on the Pd/SiO_2_ aerogel catalyst improved after the first cycle. Pd/SiO_2_ samples showed higher performance during the second cycle due to the removal of precursors from the silica pores in the synthesis processes and therefore better electron transfer from the Pd nanoparticles to silica due to the formation of a well-defined Pd/SiO_2_ interface, leading to enhanced presence of active oxygen species at SiO_2_ surface. A high concentration of PdO phase could be observed after the second cycle, as shown in the XPS of the catalysts that are discussed at later point [53]. CO oxidation occurs due to the formation of CO^+^ species as a result CO adsorption on Pd^0^ nanoparticles followed by the reaction with active oxygen radicals to produce CO_2_ leaving vacant O on the support surface, that gets compensated by the adsorption of oxygen gas [54]. Along with the support porosity and the Pd particle size, the outer surface Pd nanoparticles play a crucial role in CO oxidation. Although the 3% CO conversion temperature (light-off) for the samples was at relatively high temperatures, the CO oxidation performance of the Pd/SiO_2_ catalysts is evident, where a maximum CO conversion of ∼100% was achieved at 249 °C in the second cycle. The difference between the temperatures corresponding to 50% CO conversion (T_50_) and 10% CO conversion (T_10_) was 33 °C in the second cycle compared to 45 °C in the first cycle. These results demonstrate that silica aerogel-supported Pd catalysts lower the activation energy of the CO oxidation reaction. The CO oxidation performance of the catalysts at different temperatures can be explained based on three stages of operation. At low temperatures (<200 °C), a low level of CO conversion occurs since the conversion is kinetically controlled and highly dependent on oxygen adsorption [55].

At moderate temperatures (>200 °C), there is a higher catalytic performance due to the oxidation of metallic Pd and the dissociated adsorption of O_2_ and CO. Conversions are controlled by diffusion and mass transfer limitations in the SiO_2_-based porous support [56]. At higher temperatures (>250 °C), the full CO conversion stage, CO conversion is extremely high and controlled by external (gas phase) diffusion without any mass transfer limitations.

### 3.3 Moisture effect on catalytic conversion of CO

**Fig. 10:**
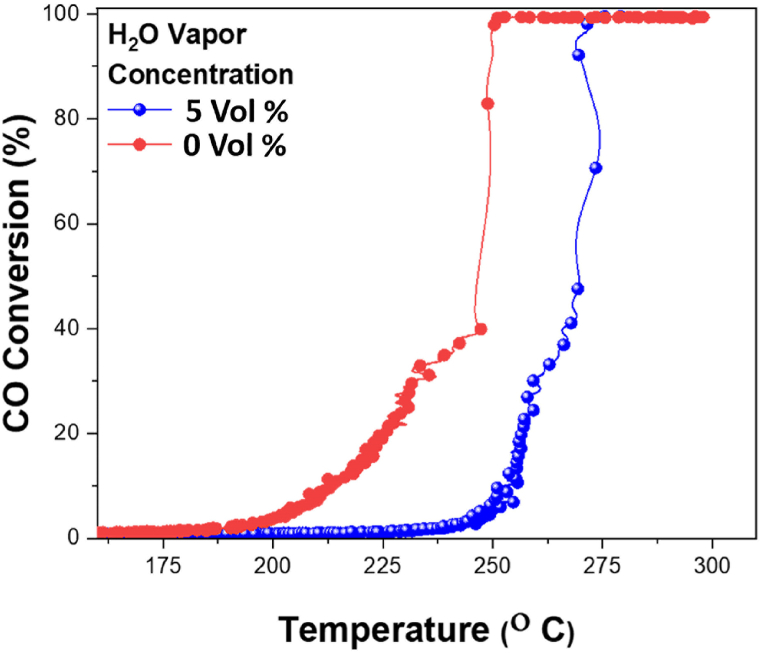
Comparison between Pd/SiO_2_ catalyst with and without water vapor at 100 cm^3^/min flow rate, 20 mg catalyst weight, and 10 °C/min heating rate.

Moisture is inevitable in applications especially at low temperatures and it is important to study the catalytic behavior of the Pd/SiO_2_ catalyst with moisture in the reaction gas. Quantity of moisture adsorbed on the catalyst determines the activity in such conditions. Low water (H_2_O) vapor content are favorable for oxidation of CO, while higher concentrations reduce catalyst activity especially for porous support materials as H_2_O molecules could potentially block the active sites [57]. To study the catalytic activity of Pd/SiO_2_ with and without moisture, 5% H_2_O vapor was introduced to the gas feed. Fig. 10 shows that the presence of moisture reduces the activity of catalysts slightly but shifts the light-off temperature to a higher temperature, which is in line with observations made by other authors [58].

### 3.4 Adsorption/desorption of CO_2_ gas onto Pd/SiO_2_

A gravimetric microbalance was used to measure adsorption/desorption Isotherms for CO_2_ onto Pd/SiO_2_ aerogel at different temperatures. Before the adsorption measurements, the samples were regenerated and initially exposed to flotation in a helium atmosphere to inspect the mass and skeleton volume of the loaded sample. These buoyancy measurements revealed that the Pd/SiO_2_ aerogel has a skeleton density of 0.1017 g/cm^3^.

**Fig. 11:**
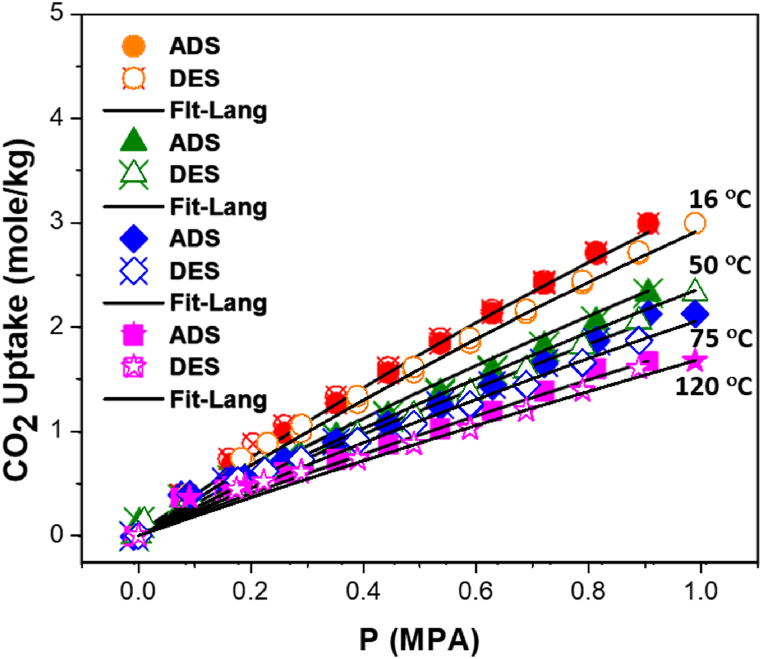
Adsorption/desorption isotherms of carbon dioxide (CO_2_) onto 5% palladium/silica (Pd/SiO_2_) aerogel at different temperatures. Empty and solid symbols refer to experimental adsorption and desorption data, and lines refer to the corresponding Langmuir correlation.

Fig. 11 shows the measured adsorption isotherms of CO_2_ at different temperatures; 16, 50, 75, and 120 °C. Overall, it revealed that the adsorption capacity of CO_2_ decreases by increasing the temperature. Furthermore, the adsorption/desorption capacity of CO_2_ gas onto Pd/SiO_2_ increases by increasing the pressure at all temperatures. The highest and lowest adsorption capacity for CO_2_ on Pd/SiO_2_ aerogel at a pressure of ∼1 MPa were 2.9 and 1.6 mol/kg at 16 and 120 °C, respectively. This trend agrees with physisorption behaviors reported in the literature [59,60]. It is to be noted that the adsorption and desorption data were almost identical, without any noticeable hysteresis. The results are compatible with previously reported results on other porous materials, such as MOFs [8,59,60]. The data suggests that Pd/SiO_2_ is effective in the adsorption of CO_2_ and exhibits a type-I adsorption isotherm, which facilitates its subsequent regeneration by pressure reduction and/or temperature increase.

The CO_2_ adsorption-desorption data were fitted collectively (at all temperatures) to the Langmuir model as described in section S2. Table 1 shows the optimum fitting parameters of the Langmuir model for the adsorption/desorption of CO_2_ gas onto 5% Pd/SiO_2_ aerogel. The lines in Fig. 11 also represent these fittings. Although small deviations from experimental data are observed at high temperatures within a small pressure range (from ∼0.1 to ∼0.2 MPa), it is observed in Fig. 11 that the Langmuir model fitting was generally in good agreement with experimental data at all temperatures, and over the whole pressure range. The accuracy of fitting the experimental data by the Langmuir adsorption isotherm model is supported by the least sum of the squared of errors (*LSSE*) and *ARE* (%) values listed in Table 1. Furthermore, the fitted monolayer saturation limit (*m*) of 18.04 mol/kg shows the very high potential of Pd/SiO_2_ to adsorb CO_2_ at higher pressures. This is also supported by the characteristic adsorption energy (*ε*), equivalent to 5711 J/mol.

**Table 1:** Optimum fitting parameters of the Langmuir adsorption isotherm of CO_2_ gas onto 5% palladium/silica (Pd/SiO_2_) aerogel.

Parameters	Value	Skeleton density (g/cm^3^)
*m* (mole/kg)	18.04	0.1017
*b*^0^ (MPa^−1^)	18.08 × 10^−3^	
ε/R (K)	686.90	
*LSSE* (mole^2^/kg^2^)	0.23	
*ARE (%)*	3.46	

Where, *m* is the monolayer adsorption capacity of the adsorbed component, $b^{0}$ is the adsorption affinity at infinite temperature, *ε* is the characteristic adsorption energy on the surface, R is the universal gas constant.

### 3.5 Moisture effect on adsorption/desorption of CO_2_ gas onto Pd/SiO_2_

**Fig. 12:**
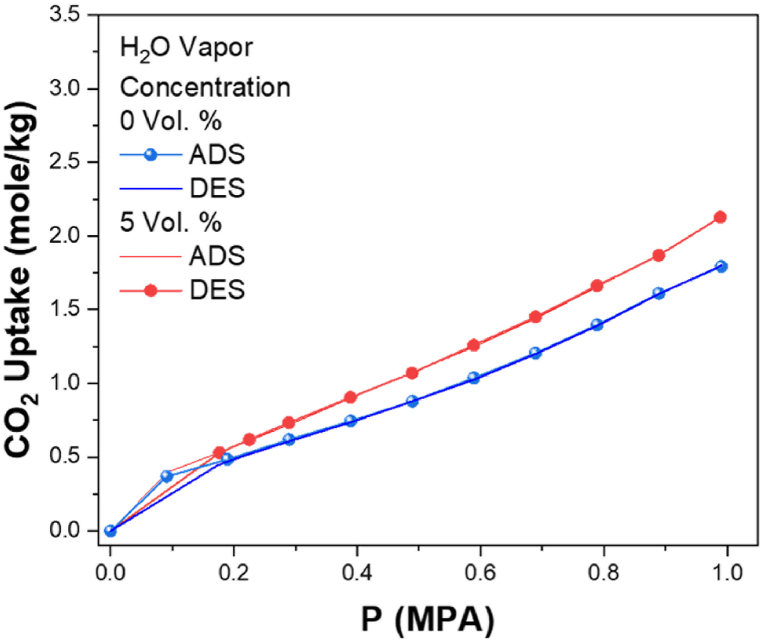
Comparison between CO_2_ adsorption/desorption on 5% Pd/SiO_2_ catalyst isotherms at 77 °C or with and without water moisture.

Moisture on the CO_2_ adsorption/desorption onto Pd/SiO_2_ was studied by adding 5 vol% H_2_O vapor into the gas feed. Fig. 12 shows that moisture improves the adsorption, where maximum CO_2_ uptake at 1 MPA increased from 1.77 to 2.12 mol/kg. This could be attributed to the lower electronegativity of Pd and the stronger interaction between the CO_2_ and the Pd nanoparticles due to the partial negative charge on the oxygen atoms and the partial positive charge on the Pd [61]. In the presence of moisture, the OH groups compete with the CO_2_ adsorption in the SiO_2_ support. However, in the presence of Pd, the stability of CO_2_ will increase due to the interaction between *CO*_*2*_^*δ*−^ with a partial positive charge on the Pd surface [61].

## 4 Conclusions

Complete oxidation of CO and capture of CO_2_ at different temperatures and pressures were evaluated over stable palladium nanoparticles supported on mesoporous silica (Pd/SiO_2_) aerogel. Pd/SiO_2_ could completely oxidize CO at relatively low temperatures (250 °C) and was efficient in adsorbing CO_2_ gas, especially at low temperatures (e.g., 16 °C). The catalysts showed excellent CO oxidation activity and could completely catalyze this reaction at low temperatures (below 300 °C).

The support’s structure and morphology, along with the oxidation state of the Pd particles, play a crucial role in its catalytic performance. The particle size, the concentration of PdO, porosity, and the Pd dispersion are important factors to consider in achieving high CO conversion. The combination of Pd with silica aerogel is crucial for forming highly active sites with abundant Pd^2+^ for low-temperature CO oxidation. Moreover, the adsorption capacity of CO_2_ on Pd/SiO_2_ was comparable to other supported sorbent materials. An application for the Pd/SiO_2_ material could be suggested in two separated CO conversions to CO_2_ and adsorption of CO_2_. The Langmuir adsorption isotherm model was used successfully to correlate the absolute experimental amounts adsorbed for CO_2_ as a function of temperature and pressure. A comparison between the CO oxidation and CO_2_ adsorption onto Pd/SiO_2_ aerogel with and without moisture showed that, although the catalytic activity was reduced slightly in moisture, the CO_2_ uptake was increased. These results indicate that Pd/SiO_2_ aerogel catalysts can provide low-cost and environmentally friendly methods for converting CO to CO_2_ and capturing CO_2_ at low temperatures.

## Author contribution statement

Rola Mohammad Al Soubaihi: Conceived and designed the experiments; Performed the experiments; Analyzed and interpreted the data; Wrote the paper. Khaled Mohammad Saoud: Contributed reagents, materials, analysis tools or data. Ahmad Awadallah-F, Ahmed Mohamed Elkhatat: Performed the experiments. Shaheen A. Al-Muhtaseb: Conceived and designed the experiments; Analyzed and interpreted the data; Contributed reagents, materials, analysis tools or data; Wrote the paper. Joydeep Dutta: Conceived and designed the experiments.

## Data availability statement

Data will be made available on request.

## Declaration of competing interest

The authors declare that they have no known competing financial interests or personal relationships that could have appeared to influence the work reported in this paper.

## Figures and Tables

**figs1:**